# Epidemiological Characteristics and Spatial-Temporal Analysis of Tuberculosis at the County-Level in Shandong Province, China, 2016–2020

**DOI:** 10.3390/tropicalmed7110346

**Published:** 2022-11-01

**Authors:** Yuqi Duan, Jun Cheng, Ying Liu, Qidi Fang, Minghao Sun, Chuanlong Cheng, Chuang Han, Xiujun Li

**Affiliations:** 1 Department of Biostatistics, School of Public Health, Cheeloo College of Medicine, Shandong University, Jinan 250101, China; 2 Shandong Public Health Clinical Center, Jinan 250101, China

**Keywords:** tuberculosis, epidemiological characteristics, spatio-temporal analysis, spatial autocorrelation

## Abstract

(1) Background: Tuberculosis (TB) is an infectious disease that seriously endangers health and restricts economic and social development. Shandong Province has the second largest population in China with a high TB burden. This study aimed to detect the epidemic characteristics and spatio-temporal pattern of reported TB incidence in Shandong Province and provide a scientific basis to develop more effective strategies for TB prevention and control. (2) Methods: The age, gender, and occupational distribution characteristics of the cases were described. The Seasonal-Trend LOESS decomposition method, global spatial autocorrelation statistic, local spatial autocorrelation statistics, and spatial-temporal scanning were used to decompose time series, analyze the spatial aggregation, detect cold and hot spots, and analyze the spatio-temporal aggregation of reported incidence. (3) Results: A total of 135,185 TB cases were reported in Shandong Province during the five years 2016–2020. Men and farmers are the main populations of TB patients. The time-series of reported tuberculosis incidence had a long-term decreasing trend with clear seasonality. There was aggregation in the spatial distribution, and the areas with a high reported incidence of TB were mainly clustered in the northwest and southeast of Shandong. The temporal scan also yielded similar results. (4) Conclusions: Health policy authorities should develop targeted prevention and control measures based on epidemiological characteristics to prevent and control TB more effectively.

## 1. Introduction

Tuberculosis (TB) is a chronic infectious disease caused by the respiratory transmission of Mycobacterium tuberculosis that can affect many organs of the body. Infection with Mycobacterium tuberculosis does not necessarily lead to morbidity but may result in clinical disease when resistance is reduced or cell-mediated metaplasia is increased [1]. Difficulties in detecting the source of infection, controlling transmission, prevention, and recurrence [2] place a heavy burden on TB control and prevention. In 2019, TB was one of the leading causes of death from infectious diseases and antibiotic-resistant infections worldwide [3]. TB is projected to be the second-leading single-source cause of death after COVID-19 in 2020. There are 842,000 new TB cases estimated in China in 2020, with an estimated TB incidence of 59 per 100,000 [4]. China has the second highest estimated TB incidence among the 30 high-TB burden countries.

Shandong Province is a coastal province in eastern China. The number of individuals that live in Shandong Province was 101,699,900 in 2021, making it the second most populous province in China. The number of reported cases of TB in Shandong Province is always at the forefront of the reported cases of legal infectious diseases in China. The situation of tuberculosis prevention and treatment in Shandong is still serious, and prevention and control work still face many problems and challenges.

Early studies have shown significant heterogeneity in the spatial distribution of TB incidence in Shandong Province with marked seasonality [5,6]. However, the latest study of spatio-temporal distribution pattern analysis in Shandong explored the distribution of TB incidence in 2016 [7]. The epidemiological characteristics of TB are gradually changing with the implementation of TB control measures and some influencing factors such as logistic, socio-economic, and meteorological factors [8,9]. The results of earlier studies may no longer be applicable. Therefore, an updated study on the incidence of TB in Shandong Province will help to prevent and control TB in a more targeted way and improve prevention and control measures [10]. An analysis at the county-level can help us to carry out more precise prevention and control efforts.

This study conducted a temporal, spatial, and spatio-temporal analysis of reported TB data at the county scale in Shandong Province from 2016–2020. Incidence patterns were summarized, and incidence hotspots, as well as high incidence time periods, were explored. It provided an updated and refined policy basis for the development of TB prevention and control strategies in Shandong Province [11,12].

## 2. Materials and Methods

### 2.1. Study Area

The study was conducted in Shandong Province. It is located on the eastern coast of China between 34°22.9′–38°24.01′ N latitude and 114°47.5′–122°42.3′ E longitude. Shandong is the second most populous province in China. The study area was divided according to the latest administrative division of Shandong Province. Shandong Province has 16 prefecture-level cities with a total of 58 municipal districts, 26 county-level cities, and 52 counties, which make up a total of 136 county-level administrative districts.

### 2.2. Data Source

Daily data on reported cases of TB in Shandong Province from 2016–2020 were obtained from the national disease reporting information system. The monthly reported incidence data for each district and county were summarized for time series analysis and spatio-temporal scan analysis. The population data for each year were obtained from the Shandong Provincial Statistical Yearbook (http://tjj.shandong.gov.cn/col/col6279/index.html, accessed on 1 September 2022.). Gender and age data were from the Sixth National Population Census of the People’s Republic of China.

### 2.3. Statistic Analysis

#### 2.3.1. Time Series Decomposition

The time series of monthly reported TB incidence data in Shandong Province for 2016–2020 were decomposed to explore temporal patterns by the Seasonal and Trend decomposition based on LOESS (STL). The STL method was developed by R. B. Cleveland, Cleveland, McRae and Terpenning in 1990 [13]. STL is a versatile and robust method for decomposing time series [14], while the LOESS method can establish nonlinear relationships. STL uses the LOESS method to decompose the time series into three main components: trend, seasonality, and residuals. STL and its plotting were implemented in the R 4.1.1 software.

#### 2.3.2. Spatial Autocorrelation Analysis

The spatial autocorrelation statistic was used to measure the degree of interdependence between data from a location and neighboring units, and was divided into global spatial autocorrelation and local spatial autocorrelation. The overall distribution characteristics of the study area can be explored by global spatial autocorrelation, and the average degree of aggregation of similar attributes within the study area can be reflected [15]. Local spatial autocorrelation can be further used to analyze the aggregation areas.

In this study, global spatial autocorrelation can be determined by the global Moran’s I index in the reported incidence of TB in 136 districts and counties in Shandong Province. The global Moran’s I index is calculated as follows [16]:

(1) $$\begin{array}{cc} & I=\frac{n}{w}\times \frac{\sum_{i=1}^{n}\sum_{j=1}^{n}w_{\mathrm{ij}}\left(x_{i}-\bar{x}\right)\left(x_{j}-\bar{x}\right)}{\sum_{i=1}^{n}{\left(x_{i}-\bar{x}\right)}^{2}}\\ & w=\overset{n}{\underset{i=1}{\sum }}\overset{n}{\underset{j=1}{\sum }}w_{ij} \end{array}$$

where n is the total number of spatial units and denotes the attribute values of the spatial unit i, the spatial unit j is the spatial weight value, and w is the sum of all. The value will be normalized to between −1.00 and 1.00 after normalization. When the Moran’s I index is positive, it means that all regions are positively correlated in space; all regions are negatively correlated in space when the Moran’s I index is negative. When the Moran’s I index is equal to 0, the regions are randomly distributed and have no spatial correlation.

Subsequently, the Getis-Ord Gi* index was used to determine the level of local spatial autocorrelation and to determine the location of cold spots or hot spots. The index is calculated as follows [17]:

(2) $$\begin{array}{c} G_{i}^{*}=\frac{\sum_{j=1}^{n}w_{\mathrm{ij}}x_{j}-\bar{x}\sum_{j=1}^{n}w_{\mathrm{ij}}}{S\sqrt{\frac{n\sum_{j=1}^{n}w_{\mathrm{ij}}^{2}-{\left(\sum_{j=1}^{n}w_{\mathrm{ij}}\right)}^{2}}{n-1}}}\\ S=\sqrt{\frac{\sum_{j=1}^{n}x_{j}^{2}}{n}-{\left(\bar{x}\right)}^{2}} \end{array}$$

We used the ArcGIS 10.7 software to calculate the global Moran’s I index and Getis-Ord Gi* index and to visualize the results.

#### 2.3.3. Spatio-Temporal Scan Analysis

The spatio-temporal scan analysis was performed using SaTScan 10.1 to explore the spatio-temporal clustering of reported TB incidence in Shandong Province from 2016 to 2020. The spatio-temporal scan statistic is a clustering method based on a dynamic cylindrical window whose radius indicates the size of the clustering region and whose height indicates the temporal extent of the clusters. The scanning statistic method was applied to the data within the study area to construct the log likelihood ratio (*LLR*) under the assumption of Poisson distribution. Then statistical tests are performed using the Monte Carlo method, and *p*-values are calculated. Larger *LLR* values and statistically significant differences indicate that the region contained under this dynamic window has a higher probability of being an aggregated region. Relative risk (*RR*) is defined as the risk inside the scan window compared to the risk outside the scan window [18].

In this study, monthly reported incidence data were used for the study, and spatio-temporal scans were performed at the county-level. The maximum spatial clustering size and the maximum temporal clustering size were set to 30% [19] and 50% [18], respectively. The clusters with the largest *LLR* values and statistically significant differences (*p*-value < 0.05) were selected as the most likely clusters, and the other clusters with smaller *LLR* values and statistically significant differences were the secondary clusters.

## 3. Results

### 3.1. Descriptive Analysis

A total of 135,185 TB cases were reported in Shandong Province during 2016–2020. A descriptive analysis of TB cases in Shandong Province in terms of gender, age, and occupation was conducted, and the results are shown in Table 1. The results showed that there were far more male TB patients than females, accounting for 70.68% of the five-year cumulative number of cases. Farmers, workers, unemployed, retirees, and students were the five main groups of TB patients, accounting for 94.00% of all cases. Farmers were the most prevalent group, with 95,517 cases (70.66%) reported over a five-year period.

**Figure 1:**
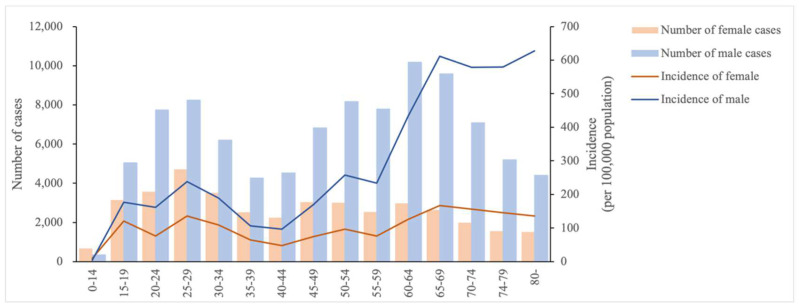
The number of cases and incidence rate of reported cases of tuberculosis by gender and age, 2016–2020.

Figure 1 showed the age and gender distribution of reported TB cases and reported incidence rates during the study period. The age of reported cases ranged from 0 to 116 years. According to the results of the Chi-square test, the reported incidence rates were lower (*p*-value < 0.05) in males than in females in the 0–14 years age group, while those in males were significantly higher **(***p*-value < 0.05) than those in females in all other age groups. In the 20–34 years age group, there was a small peak in reported incidence in both genders with a gradual increase after 45 years of age. In all age groups, there was little difference in the reported incidence rate for females, while in males the reported incidence rate increased gradually after the age of 45 years until the age of 70 years.

The reported student TB cases were mainly high school and college students (15–21 years old age group), accounting for 81.39% (5839 cases) of all student cases. The age distribution of reported incidence cases among farmers was consistent with the concentration of the entire sample in two age groups: the age group of 20–30 years and the age group of 45–70 years. The high-incidence age group of the unemployed was 20–30 years old.

### 3.2. Temporal Pattern

**Figure 2:**
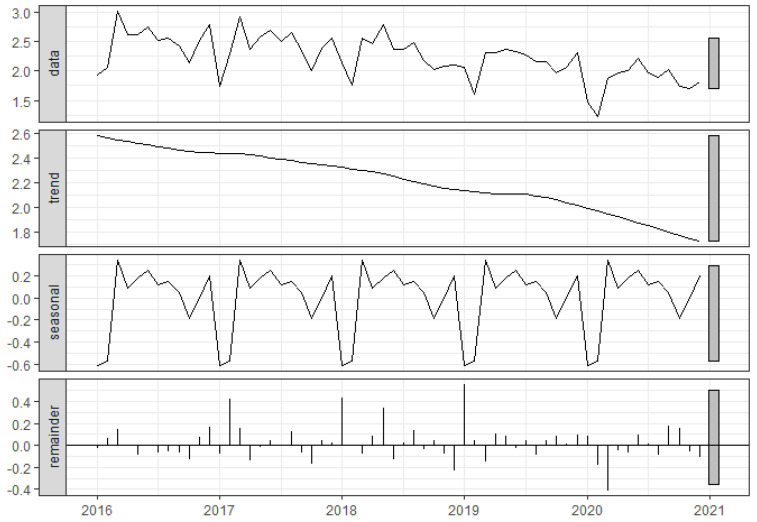
Time series decomposition of tuberculosis reported incidence from 2016 to 2020.

The time series showed a clear seasonality and long-term decreasing trend after STL decomposition (Figure 2). The reported incidence of TB in Shandong Province decreases from 30.44 (per 100,000 population) in 2016 to 22.08 (per 100,000 population) in 2020. In terms of seasonality, January and February are usually the troughs each year. They peaked in March and showed a volatile downward trend, reaching a low point in October; and then rose, reaching another small peak at the end of the year, followed by a rapid decline.

### 3.3. Spatial Distribution

**Figure 3:**
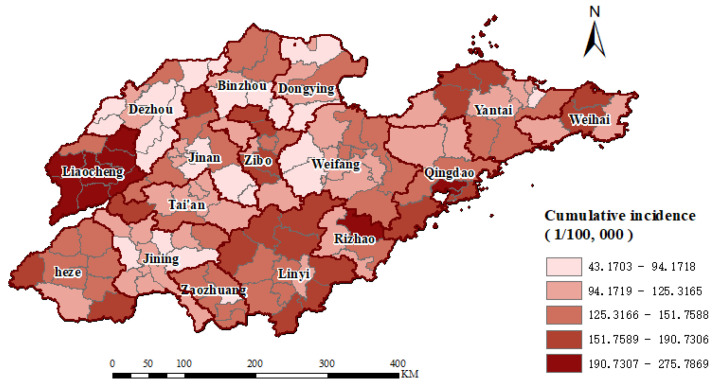
The geographical distribution of tuberculosis cumulative reported incidence in Shandong Province from 2016 to 2020.

Figure 3 showed the spatial distribution of the cumulative reported incidence rate from 2016–2020, with cases reported in each county. The five-year cumulative reported incidence rate in Shandong Province was 131.38 (per 100,000 population). Seven of the 136 counties reported incidence rates greater than 200 (per 100,000 population), five of those from Liaocheng City and two from Qingdao City. Only Lingcheng District in Dezhou City had a cumulative reported incidence rate of less than 50 (per 100,000 population).

Table 2 showed the global Moranʼs I indexes calculated based on the five-year cumulative reported incidences and annual reported incidences during this period for all districts and counties. All of them were positive and significant, which indicated a positive spatial correlation. It also suggested that the spatial distribution of tuberculosis reported incidence in Shandong Province was aggregated.

**Figure 4:**
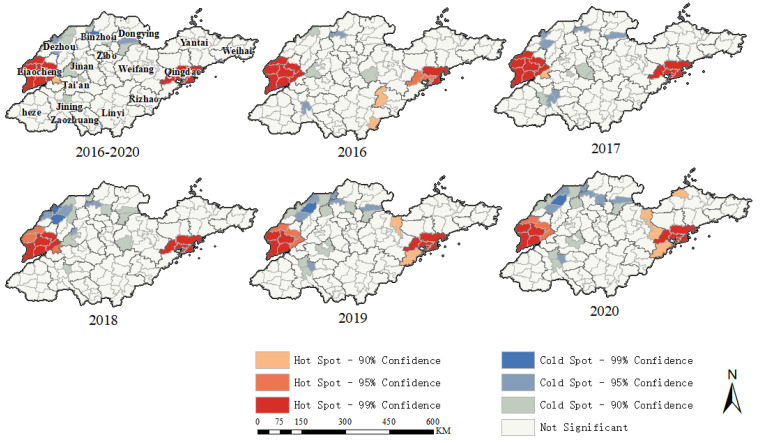
The spatial clusters of the annual and whole five-year tuberculosis reported incidence at the county-level using the Local Gi* statistic.

Figure 4 depicted the results of the local spatial autocorrelation analysis at the district and county scales for each year and the entire five-year period. The hotspots were mainly clustered in Liaocheng City as well as the southern part of Qingdao City, indicating a higher reported incidence of TB in these areas. The hotspots were relatively fixed during the five years, but there was a tendency to expand to the periphery of Qingdao City in the last two years. The distribution of cold spots was more dispersed and showed a trend of outward expansion. It developed from 5 counties and districts in 2016 to 17 counties and districts in 2020. With the development of time the cold spots were mainly concentrated in the northwest of Shandong Province.

### 3.4. Spatio-Temporal Distribution

**Figure 5:**
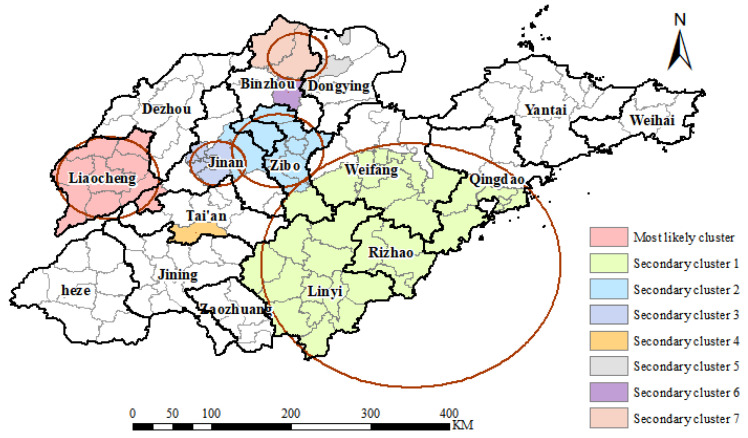
Spatio-temporal clustering of reported tuberculosis in Shandong Province from 2016 to 2020.

A spatio-temporal scanning analysis was conducted on the number of reported TB cases in Shandong Province for the period 2016–2020. Table 3 and Figure 5 showed a total of eight spatio-temporal clusters of TB identified, with one most likely cluster and seven secondary clusters. The most likely cluster included eight districts and counties in Liaocheng City and the neighboring Pingyin County in Jinan City (from 1 May 2016 to 31 October 2018). The secondary cluster 1 was the largest one which included all districts and counties in Linyi City and Rizhao City, and some neighboring districts and counties in Qingdao City, Weifang City, and Zibo City (from 1 March 2016 to 31 August 2018). The remaining secondary clusters were relatively small in spatial extent. The aggregation times corresponding to different aggregation spaces were different. This indicated that there were differences in temporal trends in TB prevalence in different regions.

## 4. Discussion

In this study, we used data from recent years to explore the epidemiological characteristics and spatio-temporal epidemiology of TB data reported in Shandong Province. In the study, we first conducted a descriptive analysis of the TB epidemic in Shandong Province, analyzed the temporal pattern of reported TB cases using the STL time series decomposition method, then studied the spatial pattern of counties using the spatial analysis method, and finally analyzed the spatial and temporal aggregation using SatSCan.

The reported incidence of TB was significantly higher in males than in females in Shandong Province, which was consistent with results reported from other regions [20]. The potential reason for this phenomenon is that men smoke and drink more alcohol than women in China, and these behaviors may lead to an increased susceptibility to TB disease. Many studies have shown that the occurrence, development, and prognosis of tuberculosis are associated with poor lifestyle habits such as smoking and alcohol consumption, which increase the risk of tuberculosis disease and death [21,22,23]. In addition, men are the main workforce in the family, engage in heavier physical work, have reduced immunity, are more likely to participate in social activities, and come into contact with people, making them susceptible to TB infection [24]. We also found that farmers accounted for 70.66% of all cases. This may be related to the living conditions of farmers such as poverty, working outside the home, high labor intensity, poor living environment, and low nutrition level [25,26]. In addition, students were also a high prevalence group of tuberculosis in this study, mainly concentrated in high school and college students. Students’ low immunity and tuberculosis infection can result from high academic pressure and insufficient exercise time during this period [27]. Moreover, group dormitory living facilitated the spread of tuberculosis [28]. We should focus on these target groups in the future control process.

Through time series analysis, we found that the reported incidence of TB in Shandong Province was on a decreasing trend. This indicated that some achievements have been made in the prevention and treatment of TB in recent years. We also found a clear seasonality in the reported incidence of TB. The reported incidence in January and February was the lowest of the year, which may be related to the patient’s visiting habits. The Spring Festival is the most important holiday in China and usually occurs in January or February. Due to customary reasons, patients usually do not choose to seek medical treatment during the Spring Festival. In addition, the largest population movement in China during the year occurs during the Spring Festival [29]. Therefore, these “Spring Festival effects” may cause the reported incidence rate to rise to its highest value in March. However, in contrast to some studies, there were two peak epidemic periods in spring and winter per year in Shandong Province. Studies of the mainland of China [30], Yunnan province [31], and Jiangsu Province [32] showed a single peak in spring or summer. They also had an upward trend in these areas during the winter, but the increase was small. This may be due to the temperature and indoor heating. Shandong Province is located in northern China, where temperatures are relatively low at the end of the year, and Shandong Province has indoor heating [33]. The corresponding decrease in outdoor activities and window openings may lead to enhanced transmission of Mycobacterium tuberculosis. High indoor population density and poor ventilation allow for a higher concentration of pathogenic bacteria in the air, leading to an increased chance of transmission of Mycobacterium tuberculosis [34]. Low temperatures can also cause low immunity and reduced serum vitamin D synthesis [35]. These can affect the development of TB [36,37]. Furthermore, it has been demonstrated that winter heating affects TB incidence not only by affecting human activity but also by increasing air pollution [38].

This study’s global spatial autocorrelation results showed that the reported incidence of TB in Shandong Province had a significant spatial clustering distribution. Further local spatial autocorrelation analysis showed that TB reported incidence hotspots in Shandong Province were mainly concentrated around Liaocheng and Qingdao cities. However, there were some differences in the spatial distribution of TB incidence in Shandong Province in the two earlier studies. In the 2005–2016 study [7], Linyi, Tai’an, Heze, and Liaocheng cities had the highest incidence. However, in the current study, high prevalence areas have changed and were mainly concentrated in Liaocheng. Areas with high incidence of TB in Shandong Province from 2005 to 2014 were mainly concentrated in the northwest of western Shandong and southeastern Shandong [6]. With the advancement of TB prevention and control, the scope of TB incidence hotspots became smaller, and even some areas in northwestern Shandong changed from hotspots to cold spots. This might be due to the modifiable areal unit problem caused by the different spatial scales of the study. In addition to the effectiveness of early tuberculosis control efforts, a reduction in the incidence rate was seen, but the reduction was caused by different magnitudes, which alternatively may have been due to differences in detection rates across locations.

One most likely cluster and seven secondary clusters were identified in the spatio-temporal scanning analysis. Similar spatial distribution of clusters was obtained in a study that focused primarily on reported cases of TB in Shandong Province in 2015 [5]. However, the southeastern region of Shandong was the most likely cluster in their study. Over time, the southeastern part of Shandong became the second most likely cluster. In its place, Liaocheng City and its surrounding areas developed as the most likely cluster. The clustering time in our study focused on 2016 to 2018, indicating that the burden of TB in Shandong Province was gradually decreasing in recent years. Our study also concluded that the time of clustering varied in different regions. This suggested that there were differences in temporal trends in TB prevalence in different regions. So we can adopt different prevention and control strategies in the future in response to the epidemic trends in different regions. This will help to carry out more precise and efficient prevention and control.

This study provides new information on the reported incidence of tuberculosis in Shandong Province, which can help the Center for Disease Control and Prevention (CDC) identify high reported incidence areas, time periods, and populations. The CDC can strengthen targeted prevention and control measures and develop more effective future strategies for tuberculosis control, such as BCG vaccination for high-risk, enhanced tuberculosis health education, and improved health insurance policies.

This study still had some limitations. Firstly, this study was mainly based on county-scale analysis, but counties are not the smallest administrative units and further research at a finer spatial scale can be considered. Secondly, the data used in this study were the reported incidence data of TB, which may differ from the actual incidence data, and there is a possibility of underestimation. Finally, TB is a multifactorial disease and the effects of, for example, socioeconomic [39], climatic [40], air pollution [41], and personal hygiene practices on the incidence of TB were not considered in this study.

## 5. Conclusions

This study explored the epidemic characteristics and spatio-temporal pattern of TB reported incidence in Shandong Province from 2016 to 2020. It also explored the spatial distribution as well as the spatio-temporal clustering of TB reported incidence at the county-level. It was found that there was a long-term decreasing trend of TB reported incidence in Shandong Province, as well as significant seasonality with two peaks in the spring and winter of a year. Although the burden of tuberculosis in Shandong has decreased in recent years, the burden of TB remains high in northwestern and southeastern Shandong. This study could help identify key areas, key time periods, and key populations with high TB reported incidence and provide a reference basis for timely adjustment or improvement of prevention and control measures.

## Figures and Tables

**Table 1:** The number of reported TB cases with different demographic characteristics in Shandong Province, 2016 to 2020.

Characteristics	2016	2017	2018	2019	2020	2016–2020
Gender						
male	21,327	20,664	19,512	18,563	15,489	95,554
female	8632	8504	8062	7630	6802	39,631
**Occupation**						
farmer	22,226	21,149	19,252	17,825	15,065	95,517
unemployed	2670	2949	2867	2802	2457	13,745
worker	1302	1185	1216	1291	1072	6066
student	1178	1291	1621	1664	1420	7174
retired	885	910	941	996	836	4568
others	1698	1684	1677	1615	1441	8115
**Total**	29,959	29,168	27,574	26,193	22,291	135,185

**Table 2:** The number of reported TB cases with different demographic characteristics in Shandong Province, 2016 to 2020.

Year	Moran’s *I*	*Z*-Score	*p*-Value
2016	0.245016	3.924002	0.000087
2017	0.348460	5.532868	<0.000001
2018	0.526354	8.161494	<0.000001
2019	0.341232	5.395392	<0.000001
2020	0.336923	5.324681	<0.000001
2016–2020	0.429948	6.793433	<0.000001

**Table 3:** Significant high-rate Clusters of reported TB in Shandong Province detected by SaTScan from 2016 to 2020.

Cluster Type	Time Frame	N	*LLR*	*RR*	*p*-Value
Most likely cluster	1 May 2016–31 October 2018	9	904.75	1.72	<0.001
Secondary cluster 1	1 March 2016–31 August 2018	32	568.65	1.29	<0.001
Secondary cluster 2	1 March 2016–30 June 2018	9	92.58	1.24	<0.001
Secondary cluster 3	1 January 2019–31 January 2019	5	84.57	2.85	<0.001
Secondary cluster 4	1 July 2016–31 December 2016	1	73.76	2.34	<0.001
Secondary cluster 5	1 November 2016–31 December 2016	1	34.98	4.00	<0.001
Secondary cluster 6	1 July 2018–31 August 2018	1	21.18	2.44	<0.001
Secondary cluster 7	1 January 2016–31 December 2017	2	18.22	1.29	0.001

N: Number of Clustering areas, *RR*: Relative risk, *LLR*: Log-likelihood ratio.

## Data Availability

Restrictions apply to the availability of these data. Data was obtained from the national disease reporting information system and are available from the authors with the permission of the national disease reporting information system.
